# Lipid metabolism in cancer cells: Its role in hepatocellular carcinoma progression and therapeutic resistance

**DOI:** 10.1097/HC9.0000000000000837

**Published:** 2025-10-21

**Authors:** Tin Lok Wong, Yanshu Kong, Stephanie Ma

**Affiliations:** 1 School of Biomedical Sciences, Li Ka Shing Faculty of Medicine, The University of Hong Kong, Hong Kong SAR, China; 2 State Key Laboratory of Liver Research, The University of Hong Kong, Hong Kong SAR, China; 3 Department of Clinical Oncology, Shenzhen Key Laboratory for Cancer Metastasis and Personalized Therapy, The University of Hong Kong-Shenzhen Hospital, Shenzhen, China

**Keywords:** chemotherapy, hepatocellular carcinoma, immunotherapy, lipid metabolism, radiotherapy, targeted therapy, therapeutic resistance

## Abstract

Liver cancer, with hepatocellular carcinoma (HCC) as its predominant form, remains among the deadliest malignancies worldwide. Despite the expanding array of treatment options, current therapies benefit only a limited subset of patients. Metabolic reprogramming is a hallmark of cancer, with lipid metabolism playing a pivotal role in tumor progression, metastasis, and therapy resistance. HCC is profoundly influenced by alterations in lipid metabolic pathways, notably those involved in steatotic liver disease, a major risk factor. Key aspects such as de novo lipogenesis, lipid uptake, fatty acid oxidation, lipid peroxidation, biosynthesis of bioactive lipids, and cholesterol biosynthesis are all reprogrammed in liver cancer cells. These metabolic shifts modify the cancer cell lipidome—altering fatty acid unsaturation levels and other lipid profiles—to promote survival and resistance during therapy. Recent technological advances have deepened our understanding of dysregulated lipid metabolism in HCC. In this review, we examine how various facets of lipid metabolism contribute to HCC disease progression and resistance to standard treatments, including tyrosine kinase inhibitors, immune checkpoint inhibitors, and radiotherapy. We also explore the potential of targeting lipid metabolic pathways to enhance therapeutic efficacy and overcome resistance, highlighting dietary interventions as a promising, low-cost, low-side-effect strategy to resensitize resistant HCC cells.

## INTRODUCTION

Liver cancer, of which 85%–90% is hepatocellular carcinoma (HCC), is one of the deadliest cancer types in the world, ranking third in cancer-related deaths. In contrast to the overall decrease in mortality rate in most cancer types in the past 20 years, the mortality rate of liver cancer continues to rise.^1^ Multiple therapeutics have been approved recently and added to the list of standard of care (SoC) for HCC, including tyrosine kinase inhibitors [TKIs; lenvatinib, sorafenib, regorafenib, cabozantinib, ramucirumab (anti-VEGFR2)] and immune checkpoint inhibitors [ICIs; atezolizumab (anti-PD-L1) plus bevacizumab (anti-VEGF), tremelimumab-actl (anti-CTLA-4) plus durvalumab (anti-PD-L1), camrelizumab (anti-PD-1) plus rivoceranib (anti-VEGFR2), nivolumab (anti-PD-1) plus ipilimumab (anti-CTLA-4), durvalumab (anti-PD-L1), pembrolizumab (anti-PD-L1)]. Despite their effect in extending survival and improving the prognosis of patients, recent analyses of real-world data have reported a low overall response rate of 26%–28% for atezolizumab plus bevacizumab and 16%–23% for lenvatinib, with a median progression-free survival of less than 12 months.^2^^–^^4^ Patients who progress after atezolizumab plus bevacizumab treatment only benefit slightly from subsequent TKIs or ICIs, with an objective response rate of 7%.^5^^,^^6^ Thus, nearly 3 out of 4 patients will not benefit from the current SoC due to resistance to therapy. Preclinical and clinical studies have proposed multiple mechanisms driving primary resistance and acquired resistance toward various treatments. Yet, resensitizing cancer cells to therapeutics has not been successful in the clinic and remains unresolved.

Lipids are crucial for normal function at both the cellular and systemic levels. They include diverse classes of molecules, including fatty acids (FAs), triglycerides (TGs), phospholipids, glycolipids, and sterols. They serve as a source of energy, building blocks for cellular structures (eg, cell membrane), signaling molecules [eg, diacylglycerol (DAG) in PKC pathway], substrates for metabolites (eg, hormones, vitamins), and cytokines (eg, prostaglandin), and are an integral part of the transportation of fat-soluble nutrients around the body. Dysregulation of lipid metabolism has been recognized as the cause of metabolic syndrome leading to the development of various diseases including cancer.^7^^,^^8^ Excess accumulation of lipids in liver parenchymal cells leads to steatotic liver disease, which is one of the key risk factors for HCC development.^9^^,^^10^ Metabolic dysfunction-associated fatty liver disease (MAFLD), previously known as non-alcoholic fatty liver disease (NAFLD), is estimated to affect >25% of adults worldwide (>1.26 billion people) in 2021.^11^^–^^13^ Nearly half of all HCC patients have MAFLD (49%), and around 12% of HCC patients have MAFLD as the sole underlying liver disease.^14^ Importantly, the proportion of HCC patients with MAFLD is expected to rise in the future.^14^^,^^15^ Recent advancements in technologies—including mass spectrometry, single-cell transcriptomics, and spatial metabolomics—have significantly enhanced our understanding of the biological roles of dysregulated lipid metabolism in cancer. These innovative approaches not only elucidate the complex metabolic alterations driving tumor progression and resistance to therapeutics but also facilitate the development of targeted therapies aimed at modulating lipid metabolic pathways.

This review synthesizes the most recent evidence on how dysregulated lipid metabolism contributes to HCC progression and resistance to current SoC therapies, including radiotherapy, TKIs, and ICIs. Given the complex interactions between tumor cells, the tumor microenvironment, and distant organs such as adipose tissue, our focus centers on alterations in lipid metabolism within cancer cells that drive disease progression and therapeutic resistance. Furthermore, we explore the potential integration of lipid metabolism targeting into existing HCC treatment strategies and discuss future perspectives in this area. In addition, the emerging role of dietary interventions aimed at modulating lipid metabolism as a complementary approach will be discussed.

## DYSREGULATED LIPID METABOLISM IN HCC AND ITS ROLE IN RESISTANCE TO THERAPY

**FIGURE 1:**
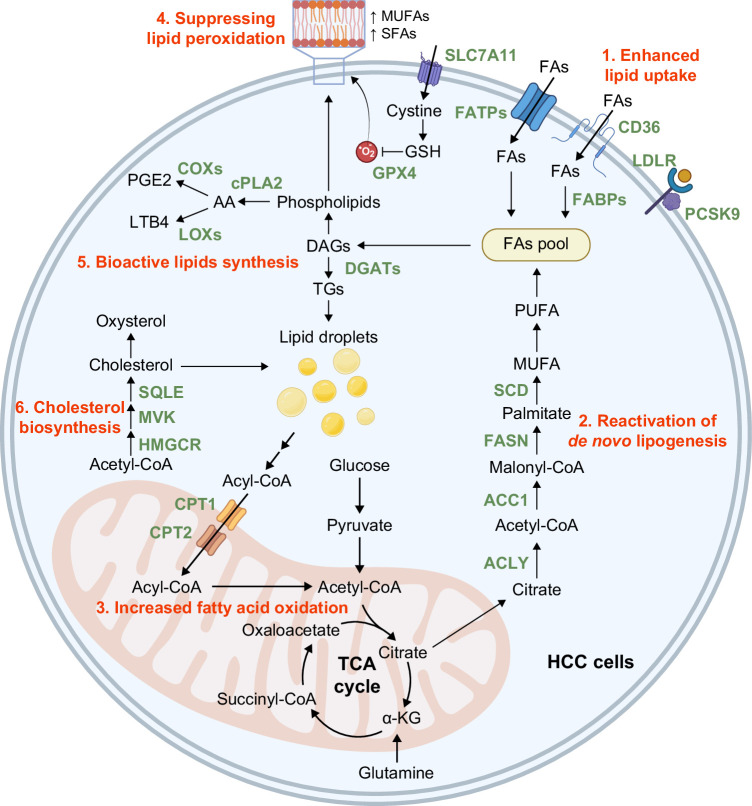
Overview of abnormal lipid metabolism in HCC. The reprogramming of lipid metabolism in cancer cells leads to the six signatures of lipid metabolism in driving cancer progression and resistance to standard of care: (1) enhanced lipid uptake, (2) reactivation of DNL, (3) increased FAO, (4) suppression of lipid peroxidation, (5) bioactive lipids synthesis, and (6) cholesterol biosynthesis. Overexpression of proteins involved in these pathways (highlighted in green) is frequently observed in HCC and has been reported to contribute to cancer progression. The products of these metabolic pathways provide energy, serve as substrates for building cellular structures, inhibit oxidative stress, and act as signaling molecules and cytokines to support the needs of cancer cells and remodel the tumor microenvironment to be pro-tumorigenic. Abbreviations: AA, arachidonic acid; DAG, diacylglycerol; DNL, de novo lipogenesis; FA, fatty acid; FAO, fatty acid oxidation; LTB4, leukotriene B4; MUFA, monounsaturated fatty acid; PGE2, prostaglandin E2; PUFA, polyunsaturated fatty acid; SFA, saturated fatty acid; TG, triglyceride.

Lipid metabolism is tightly regulated by various signaling pathways (eg, PI3K/AKT/mTOR pathway),^16^ transcription factors [eg, sterol regulatory element binding proteins (SREBPs)],^17^ and stress from the tumor microenvironment (TME) (eg, hypoxia and nutrient deprivation).^18^ Mutations in signaling pathways and overexpression of lipid metabolism-related proteins contribute to the signatures of dysregulated lipid metabolism in HCC, including (1) enhanced lipid uptake, (2) reactivation of de novo lipogenesis (DNL), (3) increased fatty acid oxidation (FAO), (4) suppressing lipid peroxidation, (5) bioactive lipids synthesis, and (6) cholesterol biosynthesis.^19^^,^^20^ A summary of dysregulated lipid metabolism in HCC is shown in Figure 1.

## ENHANCED LIPID UPTAKE

Lipid uptake is crucial for both cancer cells and normal cells. Human cells can produce only certain FAs and cannot introduce a double bond beyond the Δ9 position on FAs. Therefore, polyunsaturated fatty acids (PUFAs) are essential and must be obtained from the diet for the biosynthesis of other long-chain PUFAs.^21^ In addition, stearoyl-CoA desaturase (SCD), which introduces a double bond at the Δ9 position, requires oxygen for the conversion of saturated fatty acids (SFAs) to monounsaturated fatty acids (MUFAs). Thus, under hypoxic conditions, cancer cells cannot synthesize unsaturated FAs and must rely on the uptake of exogenous FAs to survive.^22^ Data from human HCC patients and animal models have demonstrated the importance of lipid uptake in supporting cancer growth. A high-fat diet promoted HCC formation with steatosis characteristics in diethylnitrosamine (DEN)-induced HCC mouse model.^23^ Using a spatial single-cell isotope tracing technique, ^13^C-SpaceM, Buglakova et al. observed a relative uptake of 60%–90% and thus 10%–40% DNL in mouse HCC cells, which is similar to the level found in human NAFLD patients (26% DNL).^24^^,^^25^

**FIGURE 2:**
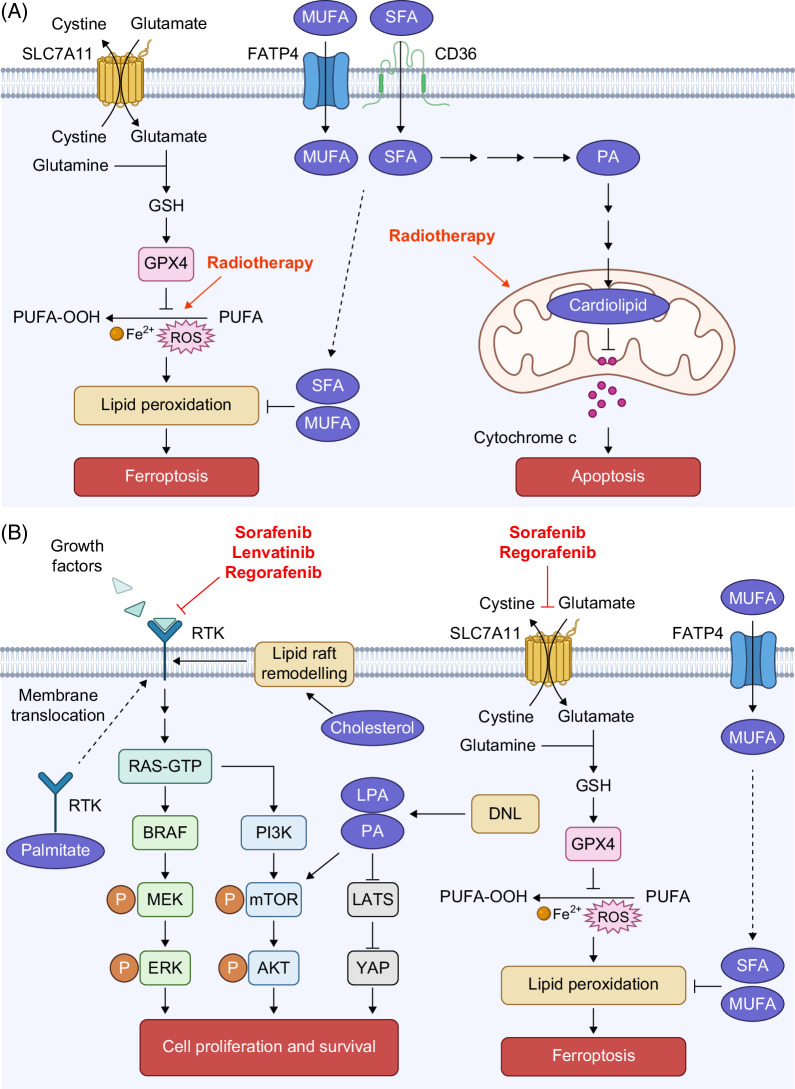
Role of dysregulated lipid metabolism on resistance to radiotherapy and tyrosine kinase inhibitors in HCC. (A) Radiotherapy-induced ROS and promoted the release of cytochrome *c* from mitochondria to drive ferroptosis and apoptosis. HCC cells enhanced the uptake of MUFA and SFA via increased expression of FATP4 and CD36 to suppress lipid peroxidation. In addition, increasing the synthesis of cardiolipid from PA maintained the integrity of mitochondria, thereby impairing the cytochrome *c*–mediated apoptosis. (B) TKIs, including lenvatinib, sorafenib, and regorafenib, inhibit RTKs to suppress proliferation and induce apoptosis of HCC cells. HCC cells reactivated DNL to activate oncogenic signaling, promoted trafficking of RTK to the cell surface via palmitoylation, and increased cholesterol biosynthesis to activate RTK to overcome suppression of RTKs by TKIs. In addition, sorafenib and regorafenib induce ferroptosis by inhibiting SLC7A11 to reduce the level of GPX4, which is a negative regulator of lipid peroxidation. HCC cells enhanced the uptake of MUFA and increased DNL of MUFA and SFA to suppress lipid peroxidation induced by TKIs. Abbreviations: DNL, de novo lipogenesis; LPA, lysophosphatidic acid; MUFA, monounsaturated fatty acid; PA, phosphatidic acid; PUFA, polyunsaturated fatty acid; RTKs, receptor tyrosine kinases; SFA, saturated fatty acid.

Uptake of FAs requires specific FA transporters, including CD36,^26^ the SLC27 family (fatty acid transport proteins, FATPs),^27^ and fatty acid-binding proteins (FABPs).^28^ CD36 is frequently upregulated in HCC and has been shown to promote cancer progression and metastasis by activating oncogenic signaling such as the Src/AKT/mTOR pathway and YAP signaling.^29^^–^^31^ Upregulation of CD36 was also observed in irradiation-resistant HCC cells after irradiation, suggesting a potential role of lipid accumulation through CD36 in driving resistance to radiotherapy^32^ (Figure 2A). Recently, Tzeng et al.^33^ developed PLT012, a neutralizing antibody targeting CD36, that showed superior effects in suppressing HCC progression in a preclinical model. Treatment with PLT012 modulated antitumor immunity by recruiting cytotoxic progenitor exhausted CD8 T cells and suppressing FOXP3+ CD4 regulatory T cells (Treg), and sensitized HCC to ICIs.^33^

FATPs facilitate the uptake of long-chain fatty acids; however, their role in HCC remains controversial. FATP4 (*SLC27A4*) was upregulated in HCC and selectively transported MUFA to protect cancer cells against lipid peroxidation and ferroptosis, thereby conferring HCC cells resistance to sorafenib^34^ (Figure 2B). On the contrary, although silencing FATP5 (*SLC27A5*) protected high-fat diet–induced liver steatosis, FATP5 deficiency has been shown to promote HCC development via activation of KEAP1/NRF2 pathway, AKT/mTOR pathway, and epithelial–mesenchymal transition (EMT).^35^^–^^40^

FABPs are proteins that bind and shuttle FAs from the membrane to intracellular destinations, for example, lipid droplets. Upregulation of FABPs, including FABP1, FABP4, and FABP5, has been observed in HCC and correlated with aggressive cancer phenotype and progression.^41^^–^^44^ FABP1 aided HCC cells to compete with T cells in the TME for uptake of long-chain FAs, especially linoleic acid, to impair T cell activation.^41^ Inhibiting LINC01116, an upstream regulator of FABP1, together with anti-PD-1 markedly reduced HCC progression and extended survival.^41^ FABP5 protected cancer cells from lipid peroxidation and ferroptosis by suppressing FAO, leading to enhanced tumor development.^42^ Inhibition of FABP5 using inhibitors SBFI-103 promoted lipid peroxidation via restoration of FAO, and increased the accumulation of macrophages expressing co-stimulatory markers (CD80 and CD86) and CD44+CD62L− effector T cells, leading to suppressed HCC progression.^42^

In addition to the expression levels of fatty acid transporters, the specific types of fatty acids being transported also influence tumor development. A high SFA diet promoted tumor formation compared with an isocaloric high PUFA diet.^45^ On the other hand, the addition of SFA palmitate has been shown to reduce tumor progression and metastasis by decreasing membrane fluidity and suppressing mTOR and STAT3 signaling pathways.^46^ The addition of ω3-PUFA eicosapentaenoic acid (EPA) to the standard diet reduced ROS level and MAPK signaling, thereby inhibiting tumor formation.^47^ Given the diversity and divergent effects of individual fatty acids, more research is needed to clarify the contribution of different FA species, dietary lipid composition, and the expression of various FA transporters in regulating tumor progression.

## REACTIVATION OF DE NOVO LIPOGENESIS

De novo lipogenesis (DNL) is a pathway that converts carbohydrates or amino acids into FAs using acetyl-CoA generated from the tricarboxylic acid (TCA) cycle by enzymes such as fatty acid synthase (FASN) and SCD.^48^^,^^49^ The newly synthesized FAs can be used for energy production via FAO during stress and production of complex lipids (eg, phospholipids) as membrane structure or signaling molecules. For example, glycerol-3-phosphate acyltransferases and lysophosphatidic acid acyltransferases synthesize lysophosphatidic acid (LPA) and phosphatidic acid (PA) respectively, which serve as building blocks for synthesis of structural lipids, such as triglyceride by diacylglycerol O-acyltransferase (DGAT), and signaling molecules, such as phosphatidylcholine.^50^ In the liver, DNL is restricted to hepatocytes in the pericentral region and is tightly regulated at the transcriptional level by transcription factors, including SREBPs and peroxisome proliferator-activated receptors (PPARs).^17^^,^^51^ However, HCC cells acquire the ability to hijack this system and reactivate DNL to support their growth and needs.

SREBP1 was regulated transcriptionally by the proto-oncogene MYC, which is frequently amplified in HCC.^20^^,^^52^^,^^53^ SREBP1 translocates to the nucleus to promote transcription of genes related to DNL, for example, *FASN*, *SCD*, and *ACACA*. Nuclear translocation of SREBP1 was promoted by the PI3K/AKT/mTOR pathway, which is activated in HCC.^16^^,^^54^^,^^55^ PPARα and PPARγ signaling drove DNL in liver and HCC, and were activated by metabolites, such as acetyl-CoA and FAs, and the AKT/mTOR pathway.^56^^,^^57^ Reactivation of DNL was also controlled at the post-transcriptional level, such as deacetylation of SREBP1 by SIRT3, acetylation of FASN by KAT8, and increased translation of ACSL family, which catalyzes the first step of incorporation of PUFA into phospholipids, by m6A modified 18S rRNA.^58^^–^^61^ These demonstrate the multiple mechanisms utilized by HCC cells to reactivate DNL.

### The role of DNL in HCC

Reactivation of DNL and the subsequent accumulation of lipids have been shown to be crucial for HCC development.^62^^–^^65^ Reduction of DNL through inhibiting maturation of SREBP1 or loss of FASN, one of the rate-limiting steps in DNL, suppressed inflammation, reduced liver steatosis, and impaired HCC formation.^66^^–^^68^ Increased levels of lysophosphatidylcholines, LPA, and triglyceride by DNL activated hepatic stellate cells to promote fibrosis and subsequent HCC development.^69^ High levels of LPA, PA, and glucosylceramide also activated YAP, AKT/mTOR, and Wnt signaling, respectively, to promote HCC development and metastasis.^70^^–^^73^ Since TKIs kill HCC cells through suppression of receptor tyrosine kinases (RTKs), activation of oncogenic signaling downstream of RTKs by PA led to resistance to lenvatinib^73^ (Figure 2B). DNL also supports the synthesis of lipids for cellular structure. Cardiolipin is a class of phospholipids found only in mitochondria. A high level of cardiolipin was observed in HCC to maintain mitochondrial integrity, thereby enhancing oxidative phosphorylation to fulfill the high energy demand for the growth of cancer cells.^62^ It also inhibited the release of cytochrome *c* from mitochondria to promote resistance to radiotherapy^74^ (Figure 2A). Although lipids are important for the growth of cancer cells, a high lipid content is also toxic to cells. Cancer cells prevent cellular damage due to the lipotoxicity via storing excess lipids as lipid droplets (LDs) and desaturation of SFAs.^75^^–^^79^ Enhanced DNL and LD formation were observed in sorafenib-resistant cells, suggesting that DNL and reducing lipotoxicity are important in driving resistance to TKIs^76^^,^^80^ (Figure 2B).

Interestingly, suppression of DNL has also been shown to promote tumor development. Nelson et al.^81^ reported that liver-specific knockout of ACC1 suppressed DNL but promoted tumor development in a DEN-induced HCC model. Inhibition of DNL led to enhanced lipid uptake via upregulation of CD36 and FATP5, potentially increasing the PUFA:SFA ratio to impair lipotoxicity, thereby promoting tumor development.^81^ However, due to the limitations of the study, definitive conclusions regarding the contribution of altered lipid profiles to tumor development remain elusive. The interplay between lipid uptake and DNL in cancer progression warrants further investigation, as understanding this relationship is crucial for the development of effective strategies to target lipid metabolism in HCC.

### The role of DNL in HCC cells in TME

**FIGURE 3:**
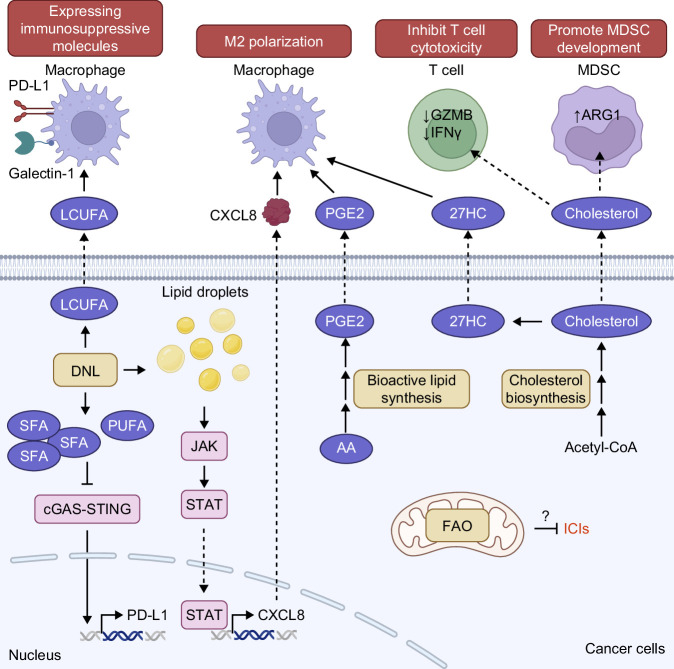
Impact of altered lipid metabolism in HCC cells on immune cell function and response to immune checkpoint blockade therapies. Reactivation of DNL modifies the lipid profile to decrease the ratio of PUFA:SFA. The low ratio of PUFA:SFA led to inhibition of the cGAS–STING pathway to downregulate PD-L1 expression and promote resistance to anti-PD-L1 therapy. FAO has also been linked with resistance to ICIs, but the mechanism is largely unexplored. In addition, DNL increased the level of LCUFA, which acted on macrophages to increase the expression of checkpoint inhibitor PD-L1 and galectin-1 in macrophages. Accumulation of lipids in HCC cells activated the JAK–STAT pathway to drive transcription of CXCL8. CXCL8 was secreted to the TME and promoted macrophage M2 polarization, which is immunosuppressive. The acquisition of M2 phenotype by macrophages was also promoted by PGE2 and oxysterol 27-hydroxycholesterol secreted by HCC cells. In addition, cholesterol secreted by HCC cells downregulated cytotoxic markers GZMB and IFNγ in T cells and increased differentiation of myeloid cells to MDSCs. These overall create an immunosuppressive TME in HCC. Abbreviations: 27HC, 27-hydroxycholesterol; AA, arachidonic acid; DNL, de novo lipogenesis; FAO, fatty acid oxidation; GZMB, granzyme B; ICIs, immune checkpoint inhibitors; IFNγ, interferon-gamma; LCUFA, long-chain unsaturated fatty acid; LPA, lysophosphatidic acid; MDSCs, myeloid-derived suppressor cells; MUFA, monounsaturated fatty acid; PA, phosphatidic acid; PGE2, prostaglandin E2; PUFA, polyunsaturated fatty acid; RTK, receptor tyrosine kinase; SFA, saturated fatty acid; TME, tumor microenvironment.

The high DNL rate in HCC created a high lipid level in the TME, which is associated with immunosuppression.^82^^–^^84^ Indeed, patients with nonviral-related HCC, such as MAFLD-related HCC, were observed to have accumulation of immunosuppressive SiglecF+ tumor-associated neutrophils and increased infiltration of exhausted immune cells, such as unconventional tissue damaging PD-1+ CD8 T cells caused by liver steatosis.^82^^,^^85^^,^^86^ Accumulation of lipids in HCC cells activated JNK/STAT signaling, thereby inducing expression of proteins that promoted polarization of immunosuppressive M2 macrophage, for example, C-X-C Motif Chemokine Ligand 8 (CXCL8), and immune checkpoint proteins, for example, programmed death-ligand 1 (PD-L1), to suppress T cell activity^82^ (Figure 3). Interestingly, overexpression of PD-L1 in HCC cells was found to induce lipid accumulation through binding of PD-L1 with EGFR and ITGB4 to activate the AKT/mTOR pathway.^87^ These studies suggest a potential positive feedback loop between DNL and PD-L1 to escape immune surveillance. In addition, HCC cells secreted long-chain unsaturated FAs to the TME, which activated macrophages in the TME via FABP5.^83^ This lipid-loaded, FABP5+ macrophage expressed PD-L1 and galectin-1 to inhibit T cell functions^83^ (Figure 3). However, DNL also suppressed PD-L1 expression.^84^ ATP citrate synthase (ACLY), which synthesizes acetyl-CoA from citrate, promoted DNL to lower PUFA:SFA ratio and prevent mitochondria damage due to lipid peroxidation.^84^ This suppressed cGAS–STING pathway to decrease PD-L1 expression and promote resistance to anti-PD-L1 treatment^84^ (Figure 3). This contradiction may possibly arise from the gene being targeted and the model used, as suppression of DNL by inhibition of FASN did not result in downregulation of PD-L1.^88^ Clinically, recent reanalysis of clinical trials data showed that MAFLD-related HCC was not responsive to ICIs.^89^^,^^90^ However, a small retrospective cohort reported that HCC with intratumor steatosis was more responsive to ICIs.^82^ Additional mechanistic studies and analysis of real-world data are needed to clarify the exact contribution of liver steatosis to response to ICIs.

## FATTY ACID OXIDATION

FAO serves as a vital energy source, yielding more ATP per carbon molecule than glucose. In addition to its role in energy production, FAO also produces citrate, which acts as a substrate for redox reactions and supports the biosynthesis of other molecules, such as nucleotides, through the folate cycle.^91^ However, the role of FAO in HCC remains controversial. Transcriptomics and proteomics analysis of HCC patients have observed increased FAO in tumor tissues.^92^^,^^93^ Wnt/β-catenin signaling–driven HCC model (loss of APC or β-catenin mutation) was addicted to FAO via activation of PPARα and increased expression of CPT1, the rate-limiting step of FAO.^94^^,^^95^ The increase in FAO was observed before the tumor developed and was important for the transition from premalignant liver to tumor tissue.^94^^,^^96^^,^^97^ Conversely, inhibition of FAO by etomoxir, a CPT1 inhibitor, impaired tumor development.^94^^,^^96^ Mechanistically, activation of FAO by NRas overexpression increased the metabolic rate of premalignant cells, leading to the generation of ROS and causing DNA damage.^96^ High FAO also supported the maintenance of tumor-initiating cells, which are linked to resistance to sorafenib.^98^ On the other hand, suppression of FAO was important for p53 mutation-driven HCC and DEN plus high-fat diet–induced HCC model.^99^^,^^100^ The discrepancy between these studies may be due to differences in etiology and genetic mutations. The differences in dysregulated lipid metabolism in HCC driven by different etiologies warrant further exploration and are discussed below. Despite the discrepancy, FAO has been shown to be critical for the survival of cancer cells during nutrient deprivation.^101^^,^^102^ CPT1 was released from ACC1 and translocated into mitochondria to enhance FAO and promote survival during nutrient deprivation.^102^ In addition, using a signature of fatty acid degradation gene to stratify human HCC patients into 3 clusters, Liu et al.^103^ discovered that HCC patients in the cluster enriched with high expression of FAO genes are less responsive to anti-PD-1, suggesting a connection between FAO and response to ICIs (Figure 3).

## SUPPRESSING LIPID PEROXIDATION

Lipids are one of the key components in a type of programmed cell death known as ferroptosis. Ferroptosis is driven by iron-dependent phospholipid peroxidation, especially the peroxidation of phospholipids containing PUFAs but not SFAs and MUFAs.^104^ This reaction is dependent on iron and is negatively regulated by GPX4, FSP1, and SLC7A11. As discussed in the previous section, HCC has high lipid uptake and DNL, leading to the accumulation of PUFAs. For example, the ACSL4 family preferentially ligated long-chain PUFA, arachidonic acid, to coenzyme A for incorporation into phospholipids and served as an important driver of ferroptosis.^104^ However, high expression of ACSL4 also promoted HCC development through increasing lipid accumulation via synthesis of phospholipids to support growth of cancer cells and enhance inflammation to promote fibrosis.^61^^,^^105^^–^^107^ Thus, HCC cells need to overcome the increase in PUFA-driven lipid peroxidation due to lipid uptake and DNL. Moreover, ferroptosis can be induced by radiotherapy and TKIs, such as sorafenib and regorafenib. Suppressing lipid peroxidation will confer HCC cells resistance to radiotherapy and TKIs. Indeed, high expression of ACSL4 was found to negatively correlate with resistance to sorafenib and ACSL4 expression can be used as prognostic marker for predicting response to sorafenib.^108^ Remodeling lipid profile to reduce the level of PUFA also helped HCC cells to overcome lipid peroxidation stress.^109^^–^^111^ This includes rewiring metabolism to change the ratio of PUFA:SFA,^109^ impairing the incorporation of PUFA into the cell membrane,^111^ and promoting the degradation of ACC1 to reduce the overall level of PUFAs.^110^ As a key enzyme for the production of MUFA, SCD is instrumental in driving resistance to sorafenib and regorafenib treatment by lowering the PUFA:MUFA ratio^112^^–^^114^ and inhibiting ER stress-induced unfolded protein response and apoptosis caused by sorafenib treatment by altering the saturation level of FAs in cancer cells^79^^,^^115^ (Figure 2B). Moreover, HCC cells with high DNL stabilized HIF1α via FASN to increase the transcription of SLC7A11, which imports cysteine to synthesize ROS scavenger glutathione, thereby inhibiting lipid peroxidation to promote HCC development,^116^ and resistance to radiotherapy^117^ and sorafenib^118^ (Figure 2). On the other hand, HCC cells promote lipid peroxidation in T cells, leading to dysfunctional, exhausted CD8 T cells.^119^^,^^120^ Uptake of iron via TFRC and oxidized low-density lipoprotein (LDL) via CD36 by CD8 T cells within the TME resulted in lipid peroxidation, activation of p38 kinase, and increased expression of exhaustion markers, such as PD-1 and TIM-3.^119^^,^^120^ In addition, lipid uptake through CD36 elevated palmitate levels, promoting palmitoylation of STAT3.^121^ This leads to overactivation of STAT3 signaling in CD8 T cells, and subsequent terminal exhuastion, which contributes to HCC progression.^121^

## SIGNALING BY BIOACTIVE LIPIDS

Lipids can be further processed to form bioactive lipids, especially eicosanoids and ceramides. Eicosanoids include a range of molecules such as prostaglandins, leukotrienes, thromboxanes, and docosahexaenoic acids, which have autocrine and paracrine effects and help to shape the TME to support tumor growth.^122^^,^^123^ However, contradictory roles of eicosanoids have been reported. High levels of prostaglandin E2 (PGE2) promoted HCC development through acting on both cancer cells^124^ and immune cells.^125^^,^^126^ PGE2 bound to prostaglandin E_2_ receptor 4 (EP4 receptor) on HCC cells to activate oncogenic PI3K/MAPK signaling and stabilize HIF2α to survive hypoxia.^124^^,^^127^ PGE2 secreted by HCC cells bound to EP4 receptors on macrophages, activating the AKT/mTOR signaling pathway and promoting their polarization to the M2 phenotype, which in turn elicited an inflammatory response that drove steatosis, CD8 T cell exhaustion, and tumor progression^125^^,^^126^ (Figure 3). Inhibition of COX2, a key enzyme for PGE2 biosynthesis, with nonsteroidal anti-inflammatory drug (NSAIDs), for example, aspirin, celecoxib, or meloxicam, sensitized cancer cells to sorafenib and interferon-α treatment in in vitro and preclinical model.^128^^,^^129^ On the contrary, LTB4 was found to suppress HCC development by activating immune cells in the TME.^130^ Mechanistically, LTB4 binds to the receptor BLT1 on cancer cells to reduce the secretion of TGF-β via JNK signaling. The low level of TGF-β in the TME decreased polarization to M2 macrophage and increased T cell infiltration, sensitizing cancer cells to anti-PD-1 treatment.^130^ These studies suggest that the inhibition of COX2 or the addition of LTB4 can potentially sensitize cancer cells to ICIs, but more research on the multifunctional role of eicosanoids is needed, given the limited studies available in HCC.

## CHOLESTEROL BIOSYNTHESIS

Cholesterol is an important component of the cell membrane, controlling its fluidity and functioning as a precursor for the synthesis of hormones. Cholesterol biosynthesis is tightly regulated by transcription factors, for example, SREBP2 and PPARs, as well as intermediate metabolites, which act as negative regulators. Several cholesterol biosynthesis-related genes are overexpressed in cancer and are correlated with a poor prognosis, for example, *SQLE*, *HMGCR*, and *MVK*.^17^^,^^131^ Epidemiological studies have shown that the use of statins, which lower cholesterol levels in the blood, reduces cancer-related mortality, suggesting an important role of cholesterol in cancer cells.^132^ However, the role of cholesterol in cancer remains controversial as several studies also have noted the tumor suppressive role of cholesterol.^133^

### Pro-tumorigenic role of cholesterol

The pro-tumorigenic role of cholesterol in HCC is well-documented. Addition of high level of cholesterol to a high-fat diet promoted HCC development by inducing severe inflammation.^134^^–^^137^ The accumulation of cholesterol in HCC cells has been shown to be critical for HCC progression and metastasis.^138^^–^^146^ The high cholesterol level in HCC cells activated oncogenic signaling, such as AKT/mTOR, EGFR, and EMT, via remodeling of lipid rafts in the cell membrane to promote HCC progression and metastasis.^145^^,^^147^ In addition, cancer cells resistant to lenvatinib or sorafenib had high cholesterol level^139^^,^^145^^,^^147^^–^^149^ (Figure 2B). Besides activating oncogenic signal, remodeled lipid rafts also upregulated drug efflux protein ABCB1, potentially exporting TKIs.^147^ Cholesterol derivatives such as oxysterol are also important for HCC progression and resistance to therapy. Mok et al.^148^ reported that a high level of 25-hydroxycholesterol activated sonic hedgehog signaling to confer HCC cells resistance to lenvatinib. Similarly, 27-hydroxycholesterol upregulated GPX4 to protect cancer cells against lipid peroxidation to support their growth,^150^ which potentially explains the mechanism by which high cholesterol level contributes to resistance to sorafenib. Besides the role of cholesterol on cancer cells, the cholesterol synthesized by cancer cells also modified the TME extensively to promote HCC progression, metastasis, and resistance to anti-PD-1 treatment by suppressing cytotoxicity of CD4 and CD8 T cells,^140^^,^^143^^,^^146^ promoting dysfunction of natural killer T (NKT) cells,^151^ inducing immunosuppressive Arg1+ myeloid-derived suppressor cells (MDSCs)^143^ and promoting polarization of M2 macrophages^150^ (Figure 3).

### Anti-tumorigenic role of cholesterol

Despite the contribution of cholesterol in modeling steatosis and HCC animal models,^134^^,^^137^^,^^152^ high cholesterol diet has also been shown to reduce tumor development in DEN-induced HCC model.^153^ In addition, high serum cholesterol level has been reported to correlate with more cytotoxic natural killer (NK) cells in healthy individuals,^153^ and predict good prognosis and lower recurrence rate following surgery in HCC patients.^154^ These suggest a potential tumor suppressive role of cholesterol in HCC. Mechanistically, cholesterol stimulated the localization of CD44 to lipid rafts in cancer cells and suppressed the interaction of CD44 with ezrin to inhibit metastasis.^154^ Accumulation of cholesterol in NK cells remodeled the lipid raft and activated NK cells to express cytotoxic perforin and granzyme B to inhibit HCC development.^153^ The differing findings regarding cholesterol’s role in HCC development may be attributed to the specific types of cholesterol involved. Cholesterol is transported with other lipids in lipoproteins, such as LDL and high-density lipoprotein (HDL), in the body. HDL-cholesterol (HDL-C) has been linked to a lower risk of cardiovascular disease, and the opposite for LDL-cholesterol (LDL-C).^155^ An increased level of non-HDL-C was also frequently observed in NASH patients.^156^ In fact, lecithin cholesterol acyltransferase (LCAT), an enzyme for esterification of cholesterol for incorporation into HDL, was downregulated in HCC and was shown to suppress HCC development by promoting hepatic uptake of HDL-C and suppressing FAO.^157^^,^^158^ Clinically, the expression of LCAT predicted response to lenvatinib.^158^ Treatment with HDL inhibited HCC development and sensitized HCC toward lenvatinib.^157^ Thus, the type of cholesterol being administered is important for determining its effect on HCC development. However, most studies lack information on HDL-C and LDL-C levels following the establishment of the model or after the intervention. A better understanding of the role of different types of cholesterol in HCC will help to translate the basic findings into the clinic. This is particularly important as inhibition of cholesterol biosynthesis with atorvastatin has been shown to enhance tumorigenesis by promoting the synthesis of bioactive lipids like PGE2.^159^

## THE RELATIONSHIP BETWEEN HCC ETIOLOGY, GENETIC MUTATIONS, AND DYSREGULATED LIPID METABOLISM

The development of HCC is complex, involving multiple interconnected factors that contribute to its onset. Moreover, the mixed genetic mutation landscape of HCC with very few dominant drivers (eg, TERT, TP53, and CTNNB1) further magnifies the heterogeneity of HCC between patients.^52^^,^^53^ Lipid accumulation is a signature of steatotic liver disease, suggesting that DNL and enhanced lipid uptake may be critical. However, MAFLD-HCC patients showed a simultaneous activation of DNL and FAO.^93^ This is potentially due to the need for a fine balance: DNL promotes steatosis, and FAO provides energy for the proliferation of cancer cells. In a high-fat diet–induced MAFLD-HCC model, overexpression of RAS accelerated tumor development via suppressing DNL and activating FAO, suggesting the balance between DNL and FAO is critical for progression from MAFLD to HCC.^96^ Hepatitis B virus (HBV) infection is one of the leading causes of HCC. Transcriptomics and lipidomics analysis of HBV-positive HCC patients showed increased DNL.^93^^,^^160^ HBx activated LXR signaling to increase transcription of SREBP1, SREBP2, and PPAR signaling activity, thereby driving DNL and tumor development.^161^^,^^162^ Mutations have been shown to important for the progression from injured liver to cancer.^163^ Administration of DEN induces DNA damage in hepatocytes and has been used, in combination with other factors, to model the development of HCC from different etiologies—such as high-fat diet for NASH and carbon tetrachloride for liver fibrosis—within a context of high mutation burden.^164^ Interestingly, DEN-induced HCC exhibits reliance on different lipid metabolism signatures compared with other models, as discussed above. This variation partly depends on the diet used; for example, a high-cholesterol diet suppressed HCC formation, whereas a combination of high-cholesterol and high-fat diet promoted tumorigenesis.^135^^,^^153^ In addition, DEN induces recurrent mutation in *HRas* and *Braf*, leading to activation of MAPK signaling pathways.^165^ These findings suggest a potential interplay between diet and genetic mutations in driving tumor development. Further research is warranted to better understand how mutations influence response to diet, especially in premalignant lesions. As discussed in the previous section, the AKT/mTOR pathway and MYC play a pivotal role in regulating DNL. Therefore, HCC driven by mutations activating the AKT/mTOR pathway and amplification of MYC showed reliance on DNL, which can be suppressed by inhibition of FASN.^62^^,^^67^^,^^72^^,^^166^^,^^167^ On the other hand, HCC driven by activation of Wnt/β-catenin signaling has been shown to rely on FAO.^94^^,^^95^ The activation of β-catenin signaling induced PPARα expression and activity to promote FAO, which provided energy for the proliferation of cancer cells.^94^ These cells were reprogrammed to synthesize more phospholipids and inhibition of FAO by etomoxir suppressed tumor development.^94^ However, loss-of-function mutation of RNF43, a negative regulator of Wnt/β-catenin signaling, activated DNL to drive HCC development.^168^ Thus, genetic alteration plays an important role in the reprogramming of lipid metabolism in HCC. Indeed, although HCC driven by loss of Pten in combination with overexpression of cMet activated DNL via activating AKT/mTOR pathway, genetic ablation of Fasn only delayed tumor development, and all mice with loss of Fasn eventually developed tumors.^167^ HCC cells without FASN were found to activate cholesterol biosynthesis and enhance lipid uptake to support tumor growth, while genetic loss of both Fasn and Srebp2 completely abolished tumor development.^167^ Collectively, these show that the reprogramming of lipid metabolism is regulated by both genetics and etiology, and metabolic plasticity plays a role in overcoming the effect of targeting lipid metabolism.

## CROSSTALK BETWEEN LIPID METABOLISM, GLUCOSE METABOLISM, AND AMINO ACID METABOLISM

The metabolism of different metabolites is interlinked. Enhanced lipid uptake activated AKT/mTOR pathway and increased palmitate level to drive glycolysis.^23^^,^^30^ Lipid metabolism, glucose metabolism, and amino acid metabolism intersect at the TCA cycle, where acetyl-CoA can be generated from the catabolism of fatty acids, glucose, and amino acids. Thus, changes in the TCA cycle lead to extensive metabolic rewiring. OGDHL converted α-ketoglutarate to succinyl-CoA in the TCA cycle and was frequently downregulated in HCC.^169^ This rewired the metabolism of glucose and glutamine to support DNL using glutamine via the reductive carboxylation instead of glucose, driving tumor progression.^169^ Metabolic plasticity allows cells to overcome a stressful environment. During nutrient starvation, HCC cells activated both DNL and FAO to survive.^102^^,^^170^ These cells substituted glutamine with extracellular citrate as a source for DNL, preserving glutamine for other metabolic pathways.^170^ Sorafenib-resistant HCC cells shifted from FAO to glycolysis to provide energy, leading to the accumulation of lipid droplets to escape mitochondrial lipotoxicity induced by sorafenib.^76^ With the shift of first-line therapy from sorafenib to lenvatinib and ICIs in recent years, understanding the consequences of these treatments in causing metabolic rewiring in cancer cells that enable resistance to therapy is important.

## POTENTIAL OF TARGETING LIPID METABOLISM TO EXPLOIT VULNERABILITY AND REVERSE THERAPY RESISTANCE IN HCC

Given the importance of lipids in resistance to cancer therapy, efforts have been made to target lipid metabolism to enhance the efficacy of currently approved therapeutics with multiple clinical trials underway (Table 1). Drugs that have been approved for use in other conditions, such as orlistat for obesity management and statins for cholesterol management, are being investigated for their use as cancer therapies. In addition, multiple drugs have been developed to target key rate-limiting enzymes involved in DNL and FAO. Here, we summarize the use of lipid metabolism inhibitors in combination with other therapeutics to overcome resistance to therapy in HCC.

**TABLE 1:** Summary of ongoing clinical trials combining drugs targeting lipid metabolism and cancer therapies

Lipid metabolism drug	Therapy combination	Cancer type	Phase	NCT number
TVB-2640	Enzalutamide	Pancreatic cancer	Phase I	NCT05743621
TVB-2640	Paclitaxel, trastuzumab, and endocrine therapy	HER2-positive breast cancer	Phase II	NCT03179904
TVB-2640	Bevacizumab	Glioblastoma	Phase III	NCT05118776
MTI-301	Progression from the standard of care	Unresectable and refractory solid cancers	Phase I	NCT06911008
Statins	Anti-PD-1/anti-PD-L1	NSCLC	Observational	NCT05636592
Statins	Post-surgery or chemoradiotherapy	Pancreatic cancer	Observational	NCT04245644
Simvastatin	Metformin and digoxin	Advanced solid cancer	Phase I	NCT03889795
Pitavastatin	Venetoclax	CML/AML	Phase I	NCT04512105
Simvastatin	Letrozole	HR-positive and HER2-negative breast cancer	Phase I	NCT05464810
Simvastatin	Carboplatin and doxorubicin	Ovarian cancer	Phase I	NCT04457089
Atorvastatin	Ezetimibe, evolocumab, and FOLFIRINOX	Pancreatic cancer	Phase I	NCT04862260
Rosuvastatin	Apatinib	Solid tumor (except GI cancer and HCC)	Phase I	NCT04428086
Atorvastatin	Temozolomide	Glioblastoma	Phase II	NCT06327451
Simvastatin	Dual anti-HER2 therapy	HER2-positive breast cancer	Phase II	NCT03324425
Lovastatin	Pembrolizumab	HNSCC	Phase II	NCT06636734
Statins	Chemotherapy or maintenance therapy	Ovarian cancer	Phase II	NCT06468254
Simvastatin	Valproic acid and AG/PAXG	Pancreatic cancer	Phase II	NCT05821556

Abbreviations: AG, Nab-paclitaxel, gemcitabine; AML, acute myeloid leukemia; CML, chronic myeloid leukemia; FOLFIRINOX, folinic acid, fluorouracil, irinotecan, oxaliplatin; GI cancer, gastrointestinal cancer; HNSCC, head and neck squamous cell carcinoma; NSCLC, non-small cell lung cancer; PAXG, Nab-paclitaxel, gemcitabine, cisplatin, capecitabine.

### Targeting DNL

FASN has long been considered a candidate therapeutic target for cancer treatment. Several inhibitors have been developed and tested in HCC, including orlistat, TVB-2640, and TVB-366. Orlistat^62^^,^^63^^,^^88^^,^^102^ and TVB-2640^88^ have effectively suppressed HCC progression in multiple preclinical models. Besides their role as monotherapy, they have been reported to work synergistically with various cancer therapies. Orlistat, TVB-2640, and TVB-3664 have also been reported to improve the efficacy of sorafenib,^102^ anti-PD-L1,^88^^,^^171^ and cabozantinib^166^^,^^172^ in HCC. A phase IIa and IIb clinical trial showed that TVB-2640 reduced steatosis in non-alcoholic steatohepatitis patients, showing its potential in targeting DNL in HCC.^173^^,^^174^

Besides FASN, ACC1 is also a top target for inhibiting DNL. ACC1 inhibitor, ND-654, worked synergistically with sorafenib to suppress the growth of HCC.^175^ In a phase IIa clinical trial, ACC1/2 inhibitor (PF-05221304) plus DGAT2 inhibitor (PF-05221304) showed promising results in suppressing DNL in the liver and reducing liver steatosis.^176^ This shows the potential of applying these inhibitors to HCC as well. Recently, there has been growing interest in developing inhibitors targeting SCD, which plays a role in promoting resistance to multiple types of cancer therapy. Several inhibitors, including aramchol, A939572, MF-438, and SSI-4 (MTI-301), have been shown to sensitize HCC cells to chemotherapy and TKIs (sorafenib and donafenib).^67^^,^^112^^,^^115^ SSI-4 (MTI-301) is set to enter a phase I clinical trial for assessing the safety and pharmacokinetics in cancer patients (NCT06911008), with other inhibitors remaining in the preclinical phase.

Despite the promising results in preclinical HCC models and patients with steatosis, there are currently no clinical trials investigating the effect of DNL inhibitors—except SSI-4—either alone or in combination with other therapeutics for HCC. This may be due to the potential adverse effects of DNL suppression in patients, which are not fully recapitulated or measured in cell lines or animal models. For example, orlistat induces weight loss in obese/overweight patients but not in animal model.^62^ However, weight loss in cancer patients, known as cachexia, is associated with worse survival in HCC patients and is an important parameter for disease management.^177^ Several studies have reported unfavorable effects of targeting SCD, FASN, or ACC1/2, including the development of resistance,^178^ promotion of metastasis^179^ and increased plasma TG and VLDL levels.^174^^,^^176^ These findings highlighted the need for careful evaluation of potential adverse side effects associated with DNL inhibition systematically, using appropriate preclinical models and during clinical trials.

### Targeting fatty acid oxidation

CPT1 regulates the transport of fatty acids into mitochondria for FAO and is the rate-limiting step of FAO. CPT1 inhibitors, such as perhexiline and etomoxir, have been shown to inhibit HCC progression.^94^^,^^96^^,^^180^ Etomoxir has also been reported to sensitize HCC cells to sorafenib.^181^^,^^182^ However, none of the FAO inhibitors have been evaluated in a clinical trial as a combination with other therapies. This is partly because of the side effects of targeting FAO. Etomoxir was found to induce liver toxicity in a phase II clinical trial, limiting its use in the clinical setting.^183^ Teglicar (ST1326), a reversible CPT1 inhibitor, is relatively safe with low liver toxicity and is a promising drug for targeting FAO in combination with other therapies.^184^ Although there are multiple hurdles to overcome for targeting FAO, the reliance on FAO in HCC driven by Wnt/β-catenin signaling, which encompasses more than 25% of HCC patients, and the enrichment of FAO genes in HCC patients unresponsive to ICIs show the clinical impact of successfully targeting FAO in HCC.

### Targeting lipid uptake

Unlike targeting DNL and FAO, limited drugs targeting lipid uptake are available. Sulfosuccinimidyl oleate (SSO) irreversibly binds to CD36 and inhibits lipid uptake. SSO enhanced the response to anti-PD-1 treatment in HCC driven by activation of β-catenin and loss of Tp53.^185^ Recently, a humanized anti-CD36 antibody PLT012 was developed and showed a superior effect in reducing HCC progression in high-fat diet–induced HCC model and sensitized HCC cells to the standard of care of HCC.^33^ It has been granted Orphan Drug Designation for liver and intrahepatic bile duct cancer by the FDA, showing the potential of PLT012 as a treatment for HCC in the future. The difficulty in targeting lipid uptake is the potential side effect, as the expression of proteins for lipid uptake, for example, FABP4 and CD36, is not restricted to cancer cells. Normal cells, such as adipocytes and endothelial cells, also express these proteins, albeit at a lower level, and more research is needed to understand the long-term effect of targeting lipid uptake at the whole-body level.

### Targeting cholesterol biosynthesis

Statins inhibit HMGCR, the key enzyme for cholesterol biosynthesis, and have been used for the treatment of dyslipidemia. Several studies have found that the use of statins can prevent the formation of HCC and improve the overall survival and disease-free survival of cancer patients.^186^^–^^188^ Lovastatin and simvastatin have also been reported to sensitize HCC cells to sorafenib and lenvatinib.^145^^,^^148^^,^^189^ Although results from preclinical studies strongly support the use of statins to circumvent therapy resistance, disappointing results were observed in several phase 2 and 3 clinical trials in HCC.^190^^–^^192^ A key factor contributing to the discrepancy between preclinical and clinical outcomes appears to be the type of statin used: lipophilic statin in preclinical studies versus hydrophilic statin in clinical trials. Epidemiological studies demonstrated a more pronounced reduction in HCC risk with lipophilic statins (eg, simvastatin, lovastatin, atorvastatin) compared with hydrophilic statins (eg, pravastatin and rosuvastatin).^187^^,^^193^ Furthermore, at doses that similarly reduce LDL-C levels, lipophilic and hydrophilic statins have been shown to modulate different set of genes in HCC cell lines.^194^ A deeper understanding of how these two classes of statins influence cellular signaling and phenotypes is essential for evaluating their therapeutic efficacy. Another potential factor is the effect of statins on modulating the levels of cholesterol in patients. Although LDL-C levels were reduced in patients treated with statins, the direction of change in HDL-C levels depended on the baseline HDL-C and TG levels.^195^ The development of a suitable preclinical model to recapture the inter-patient difference observed in patients is crucial.

### Dietary interventions

Metabolism is tightly regulated by multiple pathways, and inhibition of a single protein may lead to compensatory effects from another pathway. This may contribute to some of the contradictory results seen between in vitro and in vivo, and between preclinical and clinical trials with inhibitors targeting a single protein. Therefore, dietary intervention has also been investigated to modulate cancer lipid metabolism systematically. Calorie restriction diet, ketogenic diet and Mediterranean diet have been reported to effectively inhibit DNL and reduce steatosis in MAFLD patients and animal model.^196^^–^^198^ Thus, these diets hold the promise to reverse immunosuppressive TME and resensitize MAFLD-related HCC to ICIs as well as other SoC. Besides the diet, the composition of fatty acids in the diet is also important. A preclinical study showed that a high level of ω3-PUFAs increased the synthesis of 18-HEPE, an anti-inflammatory bioactive lipid, to suppress secretion of TNFα from macrophage thereby impairing HCC development.^199^ Since PUFAs are prone to ferroptosis, a diet high in ω3-PUFA, such as docosahexaenoic (DHA), enhanced the effect of ferroptosis inducers, for example, sorafenib, in delaying cancer progression.^200^^,^^201^ Multiple clinical trials have shown promising effect of incorporating ω3-PUFAs supplement to enhance cancer therapy.^202^^,^^203^ These studies show the potential of dietary intervention in improving response to treatment, and multiple clinical trials are underway (Table 2). However, the interaction between diet, stage of disease, genetic background of cancer, and type of therapeutics used influences the outcome of dietary intervention. Compliance with the intervention, confounding by the patients’ diet, and the low sample sizes in the current clinical trial hinder the translation to use in the clinic. Despite concerns, dietary intervention is a promising approach due to its affordability, minimal side effects, and potential to address cancer-induced cachexia.^204^ Research involving large sample sizes (exceeding 100 participants per group) and meticulous control of confounding variables will be instrumental in clarifying the impact of dietary interventions on enhancing cancer therapy outcomes.

**TABLE 2:** Summary of ongoing clinical trials combining diet intervention and cancer therapies

Diet intervention	Therapy combination	Cancer type	Phase	NCT number
Calorie-restricted diet	Post chemotherapy	Breast and prostate cancer	NA	NCT01802346
Fasting mimicking diet	Chemotherapy	Ovarian cancer	NA	NCT05921149
Fasting mimicking diet	Chemotherapy	Gynecologic malignancies	NA	NCT06376604
Fasting mimicking diet	Post PTA, TACE, or TARE	Hepatocellular carcinoma	NA	NCT06824974
Ketogenic diet	ICIs	Metastatic melanoma and kidney cancer	NA	NCT06391099
Mediterranean diet	Surgery	Prostate cancer	NA	NCT04985565
Omega-3-PUFA (DHA and EPA)	Post chemotherapy, TKIs, or radiotherapy	ERPR negative breast cancer	NA	NCT02295059
Ketogenic diet	Letrozole	ER-positive breast cancer	Phase I	NCT03962647
Ketogenic diet	ICIs	Melanoma, cSCC, RCC	Phase I/II	NCT06896552
Fasting mimicking diet	Chemotherapy plus immunotherapy	Triple negative breast cancer	Phase II	NCT06831955
Fasting mimicking diet	Chemotherapy plus immunotherapy	Triple negative breast cancer	Phase II	NCT05763992

Abbreviations: cSCC, cutaneous squamous cell carcinoma; DHA, docosahexaenoic acid; EPA, eicosapentaenoic acid; ER, estrogen receptor; ERPR, estrogen receptor and progesterone receptor; ICI, immune checkpoint inhibitor; PTA, percutaneous tumor ablation; RCC, renal cell carcinoma; TACE, transarterial chemoembolization; TARE, transarterial radioembolization; TKI, tyrosine kinase inhibitor.

## CONCLUSIONS AND FUTURE PERSPECTIVES

Multiple studies have highlighted the role of dysregulated lipid metabolism in driving cancer progression and resistance to therapy. Promising results from preclinical trials using animal models indicate the potential of targeting lipid metabolism to circumvent resistance to therapy. Dietary intervention is of particular interest as it is generally safe, low-cost, and may provide benefits beyond suppression of tumor progression, for example, reducing the risk of cardiovascular disease. However, the detailed mechanism by which dysregulated lipid metabolism contributes to therapeutic resistance is mostly unknown. For example, how does an increase in PUFAs incorporation into phospholipids promote drug resistance? In addition, the interplay between different dysregulated lipid metabolism signatures is relatively unknown. The shift between signatures may impact the success of targeting a single signature. Also, the interaction between the etiology of HCC and the genetic mutation of cancer cells in dictating the dysregulated lipid metabolism signatures is not well-documented. For example, MAFLD-HCC favors DNL while β-catenin mutation-driven HCC relies on FAO. Does targeting lipid metabolism result in different responses in MAFLD-HCC patients with or without β-catenin mutation? Moreover, the systemic effect of the intervention is poorly understood. How does the body respond to inhibition in the long term? Will there be a compensatory mechanism to increase circulating lipids when FAO is suppressed? Finally, there is a lack of clinical trials in testing the effect of combining targeting lipid metabolism with other therapies. This is partly due to the lack of inhibitors or interventions that are safe and effective for clinical trials. Further research to identify the other key enzymes as candidates and the development of novel techniques for the inhibition of lipid metabolism are warranted. While our understanding of lipid metabolism in HCC is still in its early stages, targeting lipid metabolic pathways to enhance the effectiveness of SoC holds significant promise as a transformative breakthrough.
